# The allosteric behavior of Fur mediates oxidative stress signal transduction in *Helicobacter pylori*

**DOI:** 10.3389/fmicb.2015.00840

**Published:** 2015-08-19

**Authors:** Simone Pelliciari, Andrea Vannini, Davide Roncarati, Alberto Danielli

**Affiliations:** Department of Pharmacy and Biotechnology (FaBiT), University of Bologna, Bologna, Italy

**Keywords:** ferric uptake regulator, oxidative stress, antibiotic resistance, allosteric regulation, redox regulation, metalloproteins, metal homeostasis, transcriptional regulation

## Abstract

The microaerophilic gastric pathogen Helicobacter pylori is exposed to oxidative stress originating from the aerobic environment, the oxidative burst of phagocytes and the formation of reactive oxygen species, catalyzed by iron excess. Accordingly, the expression of genes involved in oxidative stress defense have been repeatedly linked to the ferric uptake regulator Fur. Moreover, mutations in the Fur protein affect the resistance to metronidazole, likely due to loss-of-function in the regulation of genes involved in redox control. Although many advances in the molecular understanding of HpFur function were made, little is known about the mechanisms that enable Fur to mediate the responses to oxidative stress. Here we show that iron-inducible, apo-Fur repressed genes, such as *pfr* and *hydA*, are induced shortly after oxidative stress, while their oxidative induction is lost in a *fur* knockout strain. On the contrary, holo-Fur repressed genes, such as *frpB1* and *fecA1*, vary modestly in response to oxidative stress. This indicates that the oxidative stress signal specifically targets apo-Fur repressed genes, rather than impairing indiscriminately the regulatory function of Fur. Footprinting analyses showed that the oxidative signal strongly impairs the binding affinity of Fur toward apo-operators, while the binding toward holo-operators is less affected. Further evidence is presented that a reduced state of Fur is needed to maintain apo-repression, while oxidative conditions shift the preferred binding architecture of Fur toward the holo-operator binding conformation, even in the absence of iron. Together the results demonstrate that the allosteric regulation of Fur enables transduction of oxidative stress signals in *H. pylori*, supporting the concept that apo-Fur repressed genes can be considered oxidation inducible Fur regulatory targets. These findings may have important implications in the study of *H. pylori* treatment and resistance to antibiotics.

## Introduction

*Helicobacter pylori* is an obligate microaerophilic human pathobiont that colonizes the gastric mucosa of half of the world’s population. Infections can persist asymptomatically for the lifespan of the host. However, in many cases the colonization of *H. pylori* constitutes a major cause of acute and chronic gastritis, gastric and duodenal ulcer diseases, as well as gastric cancer (Salama et al., 2013). The microaerophilic nature renders the bacterium highly vulnerable to oxygen toxicity originating from the aerobic environment and endogenous sources of reactive oxygen species (ROS; Hazell et al., 2001). In addition, *H. pylori* has to counter exogenous sources of ROS derived by the inflammatory response of the gastric epithelium (Bagchi et al., 1996), eventually leading to the oxidative burst of infiltrating macrophages and neutrophils (Ramarao et al., 2000). As such, the bacterium has adapted to neutralize noxious oxigen species by expressing a rich repertoire of antioxidant factors and enzymes (Wang et al., 2006). Even though many advances have been made in understanding their molecular function and regulation, it is less clear how the oxidative signal is transduced by the bacterium to provide the coordinated responses to counteract the oxidative damage. In fact, the *H. pylori* genome lacks annotated orthologs of potential oxidative stress regulators involved in the transcriptional control of antioxidant proteins, such as OxyR, PerR, OhrR, or SoxRS (Dubbs and Mongkolsuk, 2012). Beside the post-transcriptional regulator CsrA (Barnard et al., 2004), the DNA binding (HP0119) and repair (MutS) proteins (Wang et al., 2005; Wang and Maier, 2015), and the essential orphan response regulator HsrA (HP1043), for which a role in the oxidative stress response to low levels of metronidazole (MTZ) or oxygen was recently proposed (Olekhnovich et al., 2014), the regulation of oxidative stress defenses in *H. pylori* has been repeatedly linked to the ferric uptake regulator Fur (Cooksley et al., 2003; van Vliet et al., 2004; Ernst et al., 2005; Danielli and Scarlato, 2010). Point mutations in the apo-repressed *sodB* superoxide dismutase promoter affect its Fur-dependent regulation (Carpenter et al., 2009a), while several amino acid substitutions in the coding sequence of HpFur were shown to affect resistance to MTZ (Albert et al., 2005; Choi et al., 2011), likely through the derepression of SodB (Tsugawa et al., 2011). This involvement of Fur in redox homeostasis is not surprising since the formation of hydroxyl radical species, the most reactive ROS, is intimately linked to the availability of free intracellular iron ions through the Fenton reaction (Touati, 2000). Moreover, the Fur superfamily of regulators comprises PerR and other orthologs whose roles in the transduction of oxidative stress signals have been well characterized also in other bacteria (Lee and Helmann, 2006a,b, 2007; Belzer et al., 2011; Dubbs and Mongkolsuk, 2012; Troxell and Hassan, 2013).

The Fur protein of *H. pylori* is peculiar, because of its allosteric regulation mechanism which confers the function of a transcriptional commutator switch: holo-Fur and apo-Fur each bind different regulatory elements, repressing oppositely either iron-repressible (FeOFF) or iron-inducible (FeON) gene targets, according to the intracellular iron concentration. Thereby, transcription of iron uptake genes such as *frpB* or *fecA* is repressed by holo-Fur when iron is abundant (Delany et al., 2001a; Danielli et al., 2009), while the transcription of genes encoding iron storage protein like the Pfr ferritin is induced (Delany et al., 2001b), along with genes coding for iron-cofactor proteins such as SodB and HydA (Ernst et al., 2005). On the contrary, when iron is limiting, the transcription of iron uptake genes is derepressed, together with the apo-Fur mediated repression of iron storage genes (*pfr*) and genes encoding the iron-cofactored enzymes, including *sodB* and *hydA* (Carpenter et al., 2013). Recently it has been demonstrated that this mechanism is based on the allosteric behavior of Fur which adopts different conformations and binding architectures to DNA when complexed to the iron co-factor (Agriesti et al., 2014). The peculiar apo-repression mechanism appears to rely on the presence of an additional α-helix at the N-terminus of the Fur protein, conserved in the *ε*-proteobacteria clade including *H. pylori* and *C. jejuni* (Carpenter et al., 2009b, 2013; Butcher et al., 2012; Agriesti et al., 2014). Interestingly, the point mutations in the same α-helix have been shown to strongly influence the resistance to MTZ (Choi et al., 2011), suggesting a direct link between apo-Fur repression and oxidative stress response in *H. pylori*.

It is with this background, and with the bystanding interest in the molecular basis of *H. pylori* persistence and antibiotic resistance, that we sought to further investigate the contribution of apo- and holo-Fur to the redox homeostasis of the bacterium, trying to characterize the mechanisms that enable Fur to mediate the responses to oxidative stress.

## Materials and Methods

### Bacterial Strains and Culture Conditions

*Helicobacter pylori* strains (listed in Table 1), were revitalized from glycerol stocks on Brucella broth agar plates added with 5% fetal calf serum and Skirrow’s antibiotic supplement under microaerophilic conditions in jars (Oxoid gas packs). After re-streaking on fresh plates, bacteria were cultured in a 9% CO_2_, 91% air atmosphere at 37°C and 95% humidity in a water-jacketed incubator (Thermo Forma Scientific). Liquid cultures were grown in modified Brucella broth medium supplemented with 5% fetal bovine serum in glass flasks or 25 cm^3^ sterile plastic flasks with vented cap (Corning). For transcriptional analysis, *H. pylori* planktonic cultures (OD_600_∼0.8) were treated for 10 min either with 1 mM (NH_4_)_2_Fe(SO_4_)_2_, 150 μM 2,2 Dipyridyl (Dipy) or 10 mM H_2_O_2_ before RNA extraction.

**TABLE 1:** **Bacterial strains, plasmids, and oligonucleotides**.

***Helicobacter pylori strain***	**Genotype**	**References**
G27	Clinical isolate, wild type	Xiang et al. (1995)
G27(*fur::km*)	Derived from G27 strain; bp 25 to 434 of the CDS of fur (*G27_401*) were substituted with a kanamycin resistance cassette, Km^R^	Delany et al. (2005)
**Plasmid**	**Description**	
pGEMK-F	Derivative of pGEM3Z containing a 447 bp EcoRI–PstI fragment comprising the intergenic region between *katA* and *frpB* and the 5′ end of each gene. Amp^r^	Delany et al. (2001b)
pGEMpfr	Derivative of pGemT containing the *pfr*–*G27_615* intergenic region as a 390 bp from base 699220 to base 699609 of the G27 genome, Amp^r^	Delany et al. (2001b)
**Oligos**	**Sequence**	
FrpB RT FW	TGTGAGAGGCATTGAAGACAGGCT	Agriesti et al. (2014)
FrpB RT RV	CGCCTTTGGTAACTTCCACGCTTT	Agriesti et al. (2014)
Pfr RT FW	TGCTGTTCAGCCACATACCATTGC	Agriesti et al. (2014)
Pfr RT RV	GCGCCTGAGCATAAGTTTGAAGGT	Agriesti et al. (2014)
FecA1 RT FW	AGCGTGCATGGTGTCAAAAC	This work
FecA1 RT RV	AACTTCCTTGCTCCTCCAGC	This work
HydA RT FW	GAAAGCCGCTCAATACGCAG	This work
HydA RT RV	TTGCGCGTTAGAGGGGTTAG	This work
16s RT FW	GGAGTACGGTCGCAAGATTAAA	Loh et al. (2011)
16s RT RV	CTAGCGGATTCTCTCAATGTCAA	Loh et al. (2011)

### DNA manipulations

DNA manipulations were performed with standard techniques. Restriction and modification enzymes were purchased from New England Biolabs. Preparations of plasmid DNA were carried out with a NucleoBond Xtra Midi plasmid purification kit (Macherey-Nagel).

### DNAse I Footprinting

Promoter probes were prepared as previously described (Agriesti et al., 2014). Briefly, pGEM K-F and pGEMpfr plasmids were digested with HindIII or BamHI (NEB) respectively, dephosphorylated by treatment with calf intestine phosphatase (NEB) and subsequently 5′-end labeled with [γ-^32^P]-ATP and T4 polynucleotide kinase (NEB). After a second digestion at the 3′-end of the probe, the DNA fragments were recovered by gel extraction. Recombinant His_6_-Fur was overexpressed and purified under native conditions (Delany et al., 2001b), treated with thrombin protease (10 U/mg) to remove the N-terminal histidine tag and dialyzed against Fur Footprinting buffer (10 mM Tris-Cl, pH 7.85, 50 mM NaCl, 10 mM KCl, 0.02% Igepal CA-630, 10% glycerol, 0.1 mM DTT).

The DNase I footprinting reactions were performed in 1X Fur footprinting buffer incubating approximatively 15 fmol of radiolabeled probe with different amounts of Fur protein for 20 min at room temperature, with 300 ng of sonicated salmon sperm DNA as non-specific competitor, 150 μM (NH_4_)_2_Fe(SO_4_)_2_ or 150 μM Dipy, in a final volume of 50 μl. To perform the assay in different redox conditions, DTT (1 mM or 5 mM) or H_2_O_2_ (5 mM) were added to the binding reaction.

DNAse I (Novagen) was diluted in 1X Fur FPB added with 10 mM CaCl_2_ and 5 mM MgCl_2_. Samples containing iron and DTT were digested with 0,03 U of DNase for 90 s; for all the other conditions the concentration of DNase was raised to 0,15 U.

Reactions were stopped with the addition of 140 μl of STOP buffer (192 mM NaOAc pH 5.2, 32 mM EDTA, 0.14% SDS, 64 μg/μL salmon sperm DNA), then purified and extracted. Samples were resuspended in 10 μL of formamide loading buffer, denatured at 100°C for 3 min, separated on 8 M urea-6% polyacrylamide sequencing gels and autoradiographed.

### RNA Extraction and cDNA Synthesis

Total RNA was extracted with TRI-Reagent (Sigma-Aldrich) following the manufacturer’s protocol. To remove contaminating genomic DNA, 5 μg of total RNA were treated with 1U DNAseI in 1X DNAse buffer (80 mM Hepes pH 7.5, 10 mM NaCl, 5 mM MgCl_2_, 10 mM DTT) at 37°C for 30 min in a final volume of 50 μl; then the samples were phenol/chloroform extracted, ethanol precipitated and resuspended in RNAse free mQH_2_O.

For cDNA synthesis, 1 μg of DNA-free RNA was incubated with 50 ng of random primers in a final volume of 10 μl, denatured for 5 min at 70°C and immediately chilled on ice; then 5 U of reverse transcriptase (RT-AMV, Promega), dNTPs (final concentration 1 mM each) and RT-AMV Buffer were added and the reaction was incubated for 1 h at 37°C.

### Real Time PCR

Two μL of the diluted (1:5) cDNA samples were mixed with 5 μL of 2X iTaq Universal SYBR Green Supermix (Bio-Rad) and oligonucleotides specific for the gene of interest (Table 1) in a final volume of 10 μL. Real time PCR was performed using the following cycling protocol: 50°C for 2 min, 95°C for 2 min, then 40 cycles consisting of a denaturation for 15 s at 95°C followed by 1 min at 60°C (annealing and extension step). For each real time experiment, the specificity of the reaction was checked by including a melting profile at the end of the run. Data were analyzed using the ΔΔCt method. The levels of expression of the genes of interest were normalized against the measured level of the RNA coding for the housekeeping 16S rRNA gene.

## Results

### Hydrogen Peroxide Induces Apo- Fur Repressed Genes

In order to investigate the role of Fur on the transduction of oxidative stress signals, we analyzed the transcriptional responses of Fur-regulated genes in *H. pylori* cultures exposed to hydrogen peroxide. We selected holo-Fur repressed (*frpB* and *fecA1*) and apo-Fur repressed genes (*pfr* and *hydA*) since they are oppositely regulated by Fur in virtue of an extensively described allosteric regulation mechanism responsive to iron (Delany et al., 2001b; Carpenter et al., 2013; Agriesti et al., 2014). To find the optimal conditions for the analysis, several preliminary assays were carried out (data not shown): bacterial cultures were treated with 10 to 100 mM H_2_O_2_ for 5 to 20 min, and the mRNA levels of *pfr* and *frpB* genes were assayed by RT-qPCR and compared to the untreated samples. The more reproducible results were obtained in the samples treated with 10 mM of H_2_O_2_ for 10 min. Higher concentrations of hydrogen peroxide led to erratic variations of mRNA levels, while prolonged treatments showed a return to nearly non-stressed levels after 20 min, with a bell-shaped trend of the response (data not shown).

**FIGURE 1:**
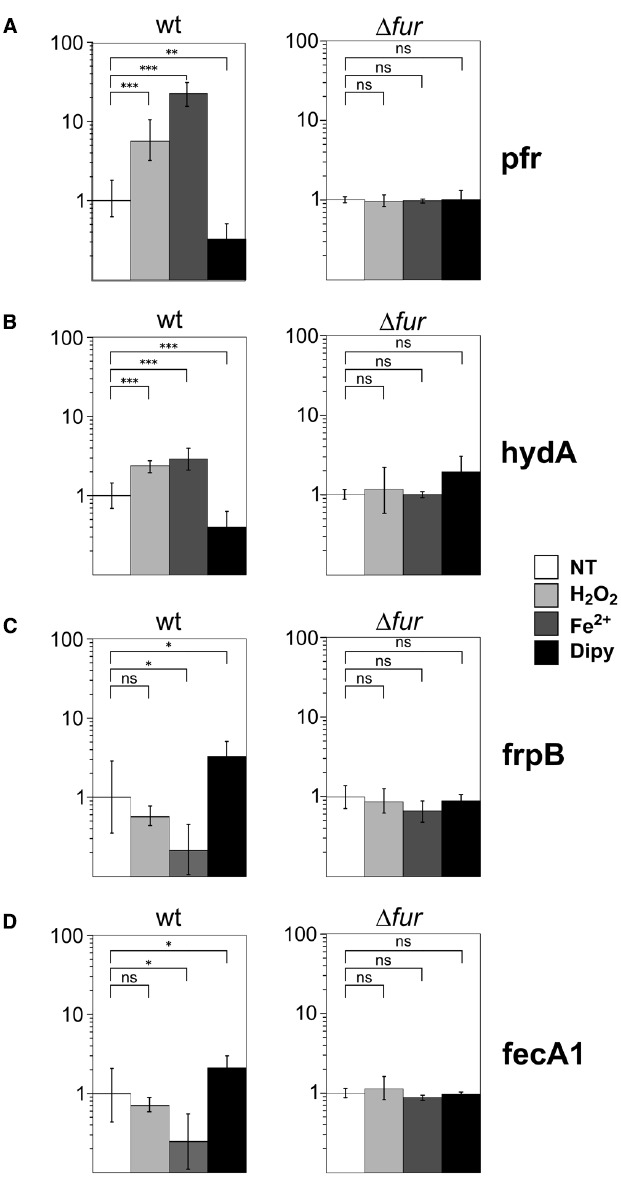
*****In vivo*** responses to oxidative stress of holo- and apo-Fur regulated genes.** Transcript levels of apo-Fur (*pfr*, *hydA*); **(A,B)** and holo-Fur (*frpB*, *fecA1*); **(C, D)** repressed genes were assayed by RT-qPCR in wild-type and Δ*fur* genetic backgrounds. Results are reported as the *n*-fold variation with respect to the untreated sample (white bars); light grey, dark grey and black bars correspond to treatments with 10 mM H_2_O_2_, 1 mM iron or 150 μM iron chelator, respectively, for 10 min. Data are reported in logarithmic scale; error bars indicate the standard deviations. The significance was calculated by a Student’s *t*-test. ns: non-significant; *p < 0.05; **p < 0.01; ***p < 0.001.

Thus, planktonic cultures of wild type and Δ*fur* strains were treated with 10 mM of H_2_O_2_ to induce the oxidative stress, or with 1 mM iron sulfate (Fe^2+^) and 150 μM iron chelator (2,2-dipyridyl; Dipy) to elicit the well-described responses occurring after iron repletion or chelation. Messenger RNA levels of genes subjected to either holo- or apo-repression were followed by RT-qPCR with results reported in Figure 1.

In the wild type strain, we observed an increase of *pfr* transcript level in response to iron and a decrease of mRNA levels upon iron chelation, as expected for an apo-Fur regulated gene. Interestingly, a sixfold increase of *pfr* mRNA level was also observed in response to the H_2_O_2_ treatment (Figure 1A). This response was lost in a Δ*fur* strain, suggesting that the observed regulation is Fur-dependent. To ascertain whether inducibility upon oxidative stress could be a conserved feature of apo-repressed Fur targets, the analysis was repeated on *hydA*, that codes for a subunit of a quinone-reactive Ni/Fe-hydrogenase, reported previously to be apo-Fur repressed as *pfr* (Carpenter et al., 2013). Consistently, the transcript levels of *hydA* were induced upon iron repletion and hydrogen peroxide treatment, and proved to be lost in a *fur* knockout background, paralleling the responses observed for *pfr* (Figure 1B).

On the other hand, holo-Fur repressed genes, such as *frpB* and *fecA1*, which are induced by the withdrawal of iron and repressed in a Fur- and iron-dependent fashion, exhibited a different, weak response to oxidative stress. In fact, the slight down-regulation upon hydrogen peroxide treatment resulted in both cases statistically non-significant (Figures 1C,D).

We conclude that apo-Fur but not holo-Fur repressed genes are responsive to hydrogen peroxide treatment, and that the oxidative stress signal mimics the effects of iron repletion on the transcription of these genes. Notably, the responses are lost in a Δ*fur* strain, suggesting that the transduction of the oxidative signal is directly or indirectly mediated by Fur.

### Hydrogen Peroxide Selectively Impairs Fur Binding to DNA

**FIGURE 2:**
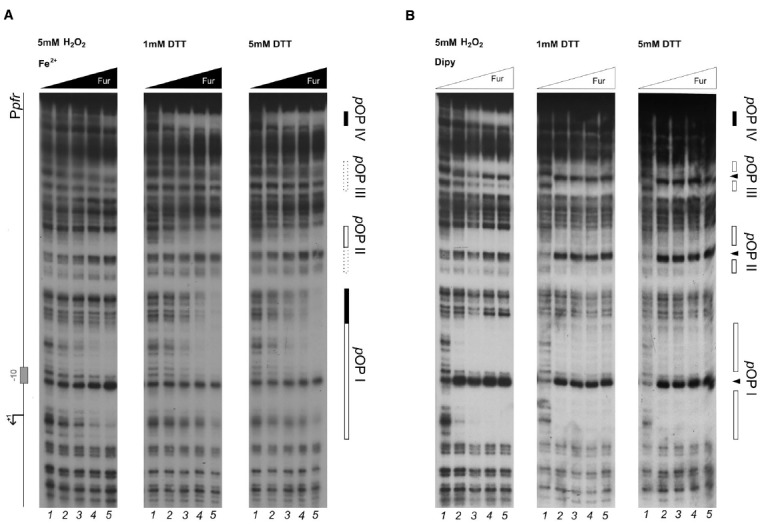
**DNase I protection patterns of Fur on the ***pfr*** promoter in reducing and oxidative conditions.** DNase I footprinting assay of Fur protein on the P*pfr* probe in presence of 150 μM of (NH_4_)_2_Fe(SO_4_)_2_ **(A)** or 150 μM Dipyridyl **(B)**. A schematic representation of the promoter region is reported on the left side of the panel. Regions corresponding to Fur operator elements are indicated by boxes: black, *holo*-Fur operators; white, *apo*-Fur operators. Arrowheads indicate hypersensitivity bands to DNase I treatment. Black and white triangles indicate increasing concentrations of Fur protein, in the presence of iron and iron chelator, respectively. The redox condition of the assay is indicated on the top of the footprinting experiment: 5 mM H_2_O_2_ (oxidative), 1 mM DTT (mildly reducing), 5 mM DTT (reducing). Lane numbers 1 to 5: 0, 29, 58, 116, and 232 nM Fur dimer.

To establish if the Fur protein mediates the oxidative stress signal response through a direct regulation, DNaseI footprinting assays were performed with the purified recombinant Fur protein and the radiolabeled P*pfr* promoter region as probe. Binding experiments were conducted under iron repletion (Figure 2A) or chelation (Figure 2B), both in reducing (5 mM DTT, 1 mM DTT) or oxidizing conditions (5 mM H_2_O_2_).

Under reducing conditions, Fur protects the P*pfr* probe in correspondence of three regions when iron is chelated, pOPI, pOPII, and pOPIII, each encasing a hypersensitive band (Figure 2B; 1 mM DTT, 5 mM DTT). These regions correspond precisely, to the three previously characterized *bona-fide* apo-operators of the P*pfr* promoter (Delany et al., 2001b). Under the same redox conditions, but in the presence of iron ions, Fur loses affinity for pOPI, pOPII, and pOPIII (Figure 2B; center and right panels), in agreement with the allosteric behavior reported recently for Fur on this promoter (Agriesti et al., 2014). Interestingly, a fourth, low-affinity, distal and iron-dependent region of protection appears (*holo*-pOPIV; Figure 2A), reported also originally by Delany and co-workers (Delany et al., 2001b).

When hydrogen peroxide is added to the reaction, the protection pattern conferred by Fur changes dramatically: the binding of apo-Fur to pOPII and pOPIII is strongly impaired, while a protection on the low-affinity holo-pOPIV operator appears, even in the absence of iron ions (Figure 2B, left panel). Similarly, hydrogen peroxide has a negative effect on Fur binding even in the presence of iron, with a general loss of affinity for all the apo-operators. Seemingly, also Fur binding to pOPIV is affected, resulting in the absence of a clear protected region on the P*pfr* probe at the highest protein concentration (Figure 2A; left panel).

### Oxidative Stress Signal Transduction on Apo-repressed Fur Targets

**FIGURE 3:**
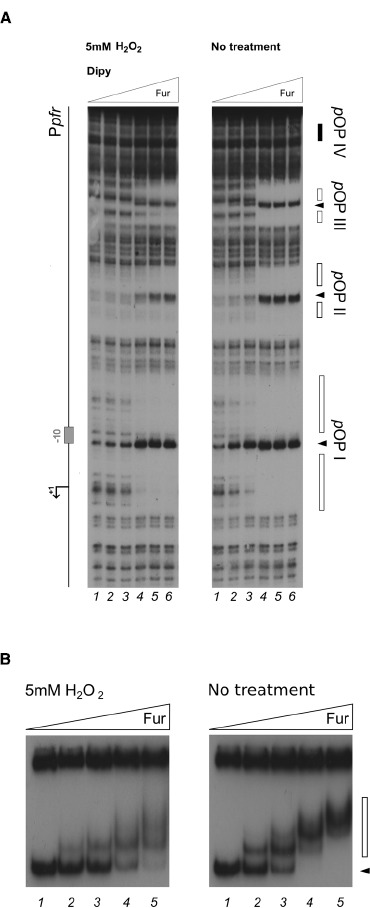
**Differential Fur binding on the apo-repressed ***pfr*** promoter in response to hydrogen peroxide. (A)** Considering the extreme affinity of the protein for the P*pfr* OPI element, we performed a footprinting at lower Fur concentrations. The protein was preincubated with the P*pfr* probe in binding buffer containing 1 mM DTT and 150 μM Dipyridyl for 10 min, then 5 mM H_2_O_2_ was added and the binding reaction was incubated for further 10 min (left panel); the control reaction (right panel) was treated with the same volume of water. Legends and symbols as in Figure 2. Lanes 1–5: 0, 0.3, 0.6, 3.3, and 8 nM Fur dimer, respectively. **(B)** EMSA performed on the *pfr* promoter probe with 1 mM DTT or 5 mM H_2_O_2_, in the presence of 150 μM Dipyridyl. Lanes 1–5: 0, 0.83, 1.7, 3.4, and 6.8 nM Fur dimer. A black arrowhead indicates the free probe, the white bar denotes the ladder generated by subsequent Fur binding events on the probe.

Since the most prominent change of Fur binding to P*pfr* in response to the oxidative signal was observed especially on the distal pOPIII and pOPIV elements, additional experiments with lowered Fur protein concentration were carried out to investigate whether also the binding to the high-affinity proximal operator elements pOPI and pOPII could be affected (Figure 3). In the footprinting experiment, an equivalent Fur protection pattern in pOPI and pOPII was observed at a fivefold higher protein concentration when hydrogen peroxide was added to the binding reaction, confirming the loss of apo-Fur binding also to the proximal high-affinity pOPI operator overlapping the core promoter region (Figure 3A). The same results where observed when the binding to the whole promoter region was assayed by EMSA (Figure 3B). These results demonstrate that oxidative conditions can impair the binding affinity of Fur to apo-operators, and promote the binding of the protein to a low affinity holo-operator (pOPIV) even in the absence of the iron-cofactor. Recalling the robust transcriptional derepression of *pfr* observed *in vivo* after hydrogen peroxide treatment, we conclude that the oxidative stress signal can be transduced in a transcriptional response on apo-repressed (FeON) Fur targets, as the direct result of a decreased binding of the Fur repressor.

### Oxidative Stress Imposes Different Binding Architectures of Fur

**FIGURE 4:**
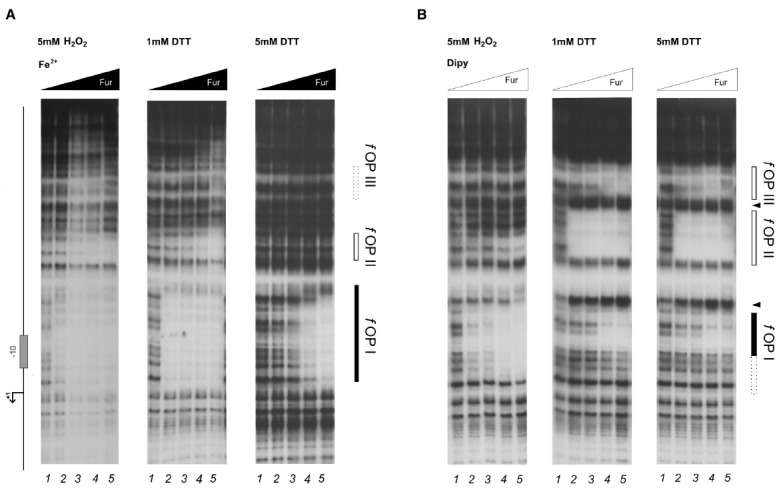
**DNase I protection patterns of Fur on the ***frpB*** promoter in reducing and oxidative conditions.** DNase I footprinting assay of Fur protein on the P*pfr* probe in presence of 150 μM of (NH_4_)_2_Fe(SO_4_)_2_ **(A)** or 150 μM Dipyridyl **(B)**. A schematic representation of the promoter region is reported on the left side of the panel. Regions corresponding to Fur operator elements are indicated by boxes: black, *holo*-Fur operators; white, *apo*-Fur operators. Arrowheads indicate hypersensitivity bands to DNase I digestion. Black and white triangles indicate increasing concentrations of Fur protein, in the presence of iron and iron chelator, respectively. The redox condition of the assay is indicated on the top of the footprinting experiment: 5 mM H_2_O_2_ (oxidative), 1 mM DTT (mildly reducing), 5 mM DTT (reducing). Lane numbers 1 to 5: 0, 29, 58, 116, and 232 nM Fur dimer.

To investigate whether a similar effect could pertain also holo-Fur targets, the footprinting analysis was extended to the P*frpB* probe, encompassing the promoter region of the iron-repressed (FeOFF) *frpB* gene (Figure 4). In agreement with the allosteric behavior of Fur (Agriesti et al., 2014), iron influenced the binding affinity of the protein on this promoter oppositely with respect to P*pfr*. Under reducing conditions, the highest affinity was observed in the presence of the iron co-factor for the proximal fOPI holo-operator, overlapping the core promoter, with a second region of protection (fOPII) appearing only at higher protein concentration upstream of fOPI (Figure 4A, center and right panels). When iron was chelated the affinity of Fur for these two elements swapped, resulting in the strong protection of the fOPII apo-operator even at the lowest Fur concentration, while binding to the fOPI holo-operator was impaired (Figure 4B; center and right panels). In addition, a third distal apo-operator appeared, fOPIII, immediately upstream of fOPII. Note also the formation of two hypersensitive bands between the three operators, indicative of modifications affecting the DNA structure.

Strikingly, the treatment with hydrogen peroxide induced distinct modifications in the protection patterns of Fur. The high-affinity binding to the fOPII and fOPIII apo-operators was abolished (Figure 4B, left panel), while the iron-dependent binding to the fOPI holo-operator was only modestly affected (Figure 4A, left panel). In addition, hydrogen peroxide provoked a Fur-dependent protection of fOPI which resembled that of the holo-protein even though the iron co-factor was chelated (Figure 4B, left panel), paralleling the effect observed for pOPIV on the P*pfr* promoter (Figure 2B; left panel). Thus, the oxidative stress signal promotes a swap in the binding architecture of Fur, impairing the binding to apo-operators and favoring to a certain extent the binding of holo-operators which require a different binding architecture of the regulator. In other words, oxidative stress induces holo-mimetic binding architectures, which appear to disfavor the binding to the apo-operators.

### Cumulative Effects of Fur Binding Affinity and Fur Binding Conformation to the *frpB* Promoter

**FIGURE 5:**
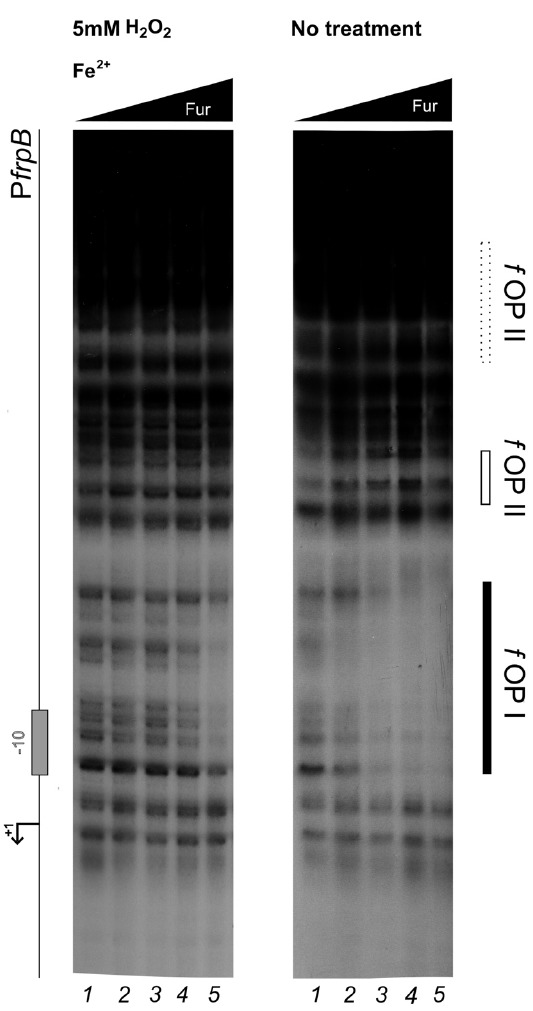
**Differential Fur binding to the ***f***OPI holo-operator in response to hydrogen peroxide treatment.** Differential protection at low Fur concentration; the protein was preincubated with the P*frpB* probe in binding buffer containing 1 mM DTT and 150 μM of (NH_4_)_2_Fe(SO_4_)_2_ for 10 min, then 5 mM H_2_O_2_ was added and the binding reaction was incubated for further 10 min (left panel); the control reaction (right panel) was treated with the same volume of water. Legends and symbols as in Figure 4. Lanes 1–5: 0, 4, 8, 17, and 34 nM Fur dimer.

The allosteric behavior of Fur induced by hydrogen peroxide suggests that the oxidative stress signal specifically targets apo-Fur repressed genes, rather than impairing indiscriminately the regulatory function of Fur. To ascertain this hypothesis and better estimate the affinity loss on holo-operators, an additional set of footprinting analyses on the P*frpB* promoter was conducted, with lowered Fur protein concentrations (Figure 5). Under reducing conditions, the protection of the fOPI holo-operator is partial at 4 nM and results in a complete protection at 8 nM Fur dimer (Figure 5; right panel, lanes 2–3). After hydrogen peroxide treatment similar protections were elicited only at 34 nM Fur dimer (left panel, lane 5). Thus we can estimate a three- to fourfold loss of holo-Fur affinity for fOPI provoked by the oxidative stress signal. However, this effect can be compensated by the gain in apo-Fur binding affinity for the same operator (Figure 4B). This evidence explains the observed responses to oxidative stress under physiological growth conditions, in which holo-Fur repressed targets such as *frpB* and *fecA1* are not significantly deregulated by oxidative stress (Figures 1C,D), while apo-Fur repressed targets (*pfr* and *hydA*) are induced by the same stimulus (Figures 1A,B).

## Discussion

*Helicobacter pylori* is highly adapted to persist in the human gastric niche and establish lifespan infections. Several lines of evidence suggest that in this environment not only *H. pylori* is exposed to low pH (Scott et al., 2007), but also to oxidative stress induced by the host inflammatory response (Ramarao et al., 2000; Ding et al., 2007). To counter these conditions, which pose a threat to the integrity of proteins and genomic DNA, the bacterium adopts a rich repertoire of antioxidant detoxification factors, including DNA binding and repairing systems (Wang et al., 2005, 2006, 2012; Wang and Maier, 2015). Clearly, these systems need to be coordinately expressed in response to an oxidative stress signal. Some regulatory mechanisms were proposed in the recent past, however, our current knowledge on how the oxidative signal is transduced to provoke a transcriptional response to ROS is still limited, mainly because *H. pylori* lacks the dedicated oxygen response regulators described in other microorganisms (Danielli et al., 2010).

**FIGURE 6:**
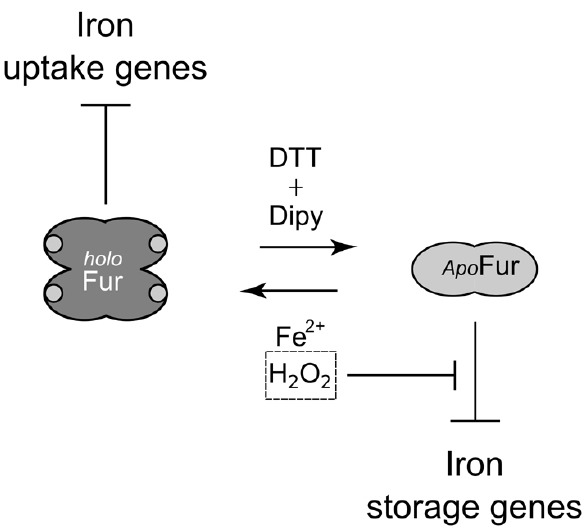
**Model of Fur behavior in response to oxidative stress.** *apo*-Fur conformation, light gray; holo-Fur conformation, dark gray. Fur represses iron uptake genes under iron-replete and oxidative conditions. *apo*-Fur targets (*pfr*) are only repressed under moderately reducing conditions. Upon an oxidative signal *apo*-Fur targets are induced, as a consequence of the allosteric behavior of Fur. Thereby, free intracellular iron can be scavenged by ferritins and metal-binding proteins, lowering the risk of iron-dependent oxidative damage that can be catalyzed by the high reactivity of this metal ion.

Here we demonstrate that HpFur can mediate the response to an oxidative signal into a specific transcriptional output. Interestingly, this transcriptional response appears to pertain mostly the apo-Fur repressed genes, i.e. genes that are inducible by free intracellular iron (FeON). Accordingly, the treatment with hydrogen peroxide mimics *in vivo* the transcriptional effect exerted by Fur in response to iron repletion on these genes (Figure 1). These results are also paralleled in the DNA binding assays *in vitro*, in which the oxidative signal confers a holo-mimetic DNA binding behavior to the transcriptional regulator. In fact, while the affinity of Fur for apo-elements as well as holo-elements is impaired in response to H_2_O_2_, the binding architecture of the regulator is switched to a conformation favoring the binding of holo-operators even in the absence of the iron co-factor (see pOPIV and fOPI; Figures 2 and 4). The evidence that Fur adopts holo-mimetic binding conformations in response to hydrogen peroxide strongly suggests that the allosteric behavior of HpFur, responsible for its function as transcriptional commutator switch (Agriesti et al., 2014), also allows for a specific transduction of the oxidative stress signal on the apo-repressed gene targets. Therefore we propose a model supporting the hypothesis that apo-Fur repressed genes can be considered oxidation inducible Fur regulatory targets (Figure 6). Consistently, many genes that are responsive to aerobic oxygen tension (Park and Lee, 2013) or ROS have also been independently listed as Fur targets in transcriptomic and ChIP-Chip analyses (Ernst et al., 2005; Danielli et al., 2006; Gancz et al., 2006)

The concept that iron-inducible apo-Fur repressed genes can be considered oxidation-inducible Fur regulatory targets (FeON = OxON) is of particular interest, especially recalling the prominent role of this metallo-regulator in the transcriptional regulatory network of the bacterium (Danielli and Scarlato, 2010). This is coherent with the intimate association of redox control and iron homeostasis, and in general with the physiology of *H. pylori*. As such, apo-Fur repressed genes such as *sodB*, which protect the cell from ROS excess, are induced by iron (FeON) and by oxygen stress (OxON), likely through the same allosteric behavior of Fur described for *pfr* and *hydA*. Similarly, the *oor* operon, which codes for an essential but oxygen-labile oxidoreductase, is induced by iron through the regulatory activity of Fur (Gilbreath et al., 2012), and is likely regulated by the allosteric behavior of Fur after an oxidative stress to compensate for oxidative inactivation. Another gene that appears to be under a resembling control is the *nifS* gene encoding a Fe-S cluster synthesis protein (Alamuri et al., 2006). In a similar fashion, *H. hepaticus* PerR is able to control both peroxide- and iron-responsive transcription of oxidative stress defenses genes (Belzer et al., 2011).

Thus, in addition to the transcriptional responses to free iron, Fur appears to cover in *H. pylori* the functions regulated by PerR in other bacteria. Interestingly, in terms of metal binding, the dimerization domain of HpFur, including the structural S1 metal-binding site, is more similar to *B. subtilis* PerR than to other Fur orthologs (Dian et al., 2011). Moreover, in HpFur two additional regulatory binding sites are found: S2 which seems to be conserved in all Fur-like proteins with some variability in the metal coordination geometry, and S3 which is present in several Fur proteins but absent in PerR orthologs. S2 is predicted to be the regulatory site responsible for the conformational changes that activate Fur for DNA binding (Dian et al., 2011), and it is interesting to recall that point mutations affecting the formation of this site impair the repression of *sodB* by HpFur (Tsugawa et al., 2011). On the contrary, the S3 site is dispensable for DNA binding but its disruption reduces the HpFur DNA-binding affinity. It was suggested that S3 may amplify the DNA-binding affinity of Fur under metal repletion (Dian et al., 2011). Thus, HpFur seems to combine the features of iron-sensing (Fur) and oxidation-sensing (PerR) Fur-superfamily members in one molecule. As such it will be worth exploring whether the additional regulatory metal binding site encompassed in the HpFur structure may be involved the oxidant sensing properties of Fur reported in this work.

Another important structural feature of HpFur is its unique N-terminal extension. It has been proposed that the N-terminal α-helix may participate in the DNA-binding activity of apo-Fur, since apo-repression could not be complemented by Fur orthologs from other species that do not contain the N-terminal extension (Miles et al., 2010). The latter is supposed to favor a V-shape conformation of HpFur in the absence of metal ion at the regulatory S2 site, as also supported by recent crystallographic studies on *Campylobacter jejuni* apo-Fur (Butcher et al., 2012). Strikingly, the mutations in this N-terminal extension greatly affect *H. pylori* resistance to MTZ, which needs to be activated by chemical reduction. Since MTZ antibiotic activity depends on the intracellular redox condition of the cell, the association of mutations in the N-terminal extension of Fur and MTZ resistance suggest a direct link between apo-repression and the Fur-dependent regulation of redox homeostasis and/or antioxidant factors in *H. pylori*.

*De facto*, although many oxidative defense genes may be regulated by other transcription factors or ncRNAs, our results support for the first time the assumption that the allosteric behavior of Fur, and apo-Fur regulation in particular, is closely associated with the transduction of oxidative stress signals, with important implications in the study of *H. pylori* treatment and resistance to antibiotics.

### Conflict of Interest Statement

The authors declare that the research was conducted in the absence of any commercial or financial relationships that could be construed as a potential conflict of interest.
